# Extracellular Vesicles Tropism: A Comparative Study between Passive Innate Tropism and the Active Engineered Targeting Capability of Lymphocyte-Derived EVs

**DOI:** 10.3390/membranes11110886

**Published:** 2021-11-18

**Authors:** Tania Limongi, Francesca Susa, Bianca Dumontel, Luisa Racca, Michela Perrone Donnorso, Doriana Debellis, Valentina Cauda

**Affiliations:** 1 Department of Applied Science and Technology, Politecnico di Torino, Corso Duca degli Abruzzi 24, 10129 Turin, Italy; francesca.susa@polito.it (F.S.); bianca.dumontel@polito.it (B.D.); luisa.racca@polito.it (L.R.); 2 Department of Neuroscience, Rehabilitation, Ophthalmology, Genetics, Maternal and Child Health, University of Genova, 16132 Genova, Italy; michela.perrone.donnorso@unige.it; 3 Department of Pediatrics, IRCCS Istituto Giannina Gaslini, University of Genova, 16128 Genova, Italy; 4 Electron Microscopy Laboratory, Center for Convergent Technologies, Istituto Italiano di Tecnologia (IIT), Via Morego 30, 16163 Genoa, Italy; doriana.debellis@iit.it

**Keywords:** extracellular vesicles, B lymphocytes, human myeloid leukemia, Burkitt lymphoma, surface labeling, cellular uptake, cell tropism, cell targeting

## Abstract

Cellular communications take place thanks to a well-connected network of chemical–physical signals, biomolecules, growth factors, and vesicular messengers that travel inside or between cells. A deep knowledge of the extracellular vesicle (EV) system allows for a better understanding of the whole series of phenomena responsible for cell proliferation and death. To this purpose, here, a thorough immuno-phenotypic characterization of B-cell EV membranes is presented. Furthermore, the cellular membrane of B lymphocytes, Burkitt lymphoma, and human myeloid leukemic cells were characterized through cytofluorimetry assays and fluorescent microscopy analysis. Through cytotoxicity and internalization tests, the tropism of B lymphocyte-derived EVs was investigated toward the parental cell line and two different cancer cell lines. In this study, an innate capability of passive targeting of the native EVs was distinguished from the active targeting capability of monoclonal antibody-engineered EVs, able to selectively drive the vesicles, enhancing their internalization into the target cancer cells. In particular, the specific targeting ability of anti-CD20 engineered EVs towards Daudi cells, highly expressing CD20 marker on their cell membrane, was proved, while almost no internalization events were observed in HL60 cells, since they did not express an appreciable amount of the CD20 marker on their plasma membranes.

## 1. Introduction

Currently, there are no more doubts that inter- and intracellular traffic mediated by vesicles represents a key role in the circulation of molecules between membrane-enclosed compartments of different secretory pathways. Vesicular transportation represents one of the main cellular activities, responsible for the regulation of the homeostatic mechanisms and cell-to-cell communication. In more detail, by avoiding referring to everything related to transport among the various compartments of the same cell, intercellular communication is central for the preservation of cell–cell homeostasis in tissues, organs, and systems of the whole human body. All cells secrete double-layered phospholipid membrane vesicles into the extracellular environment. These are ubiquitarian vesicles since they can be isolated from blood, saliva, urine, seminal fluid, breast milk, and amniotic and cerebrospinal fluid [1,2,3]. These vesicles are generically grouped as “extracellular vesicles” (EVs) [4,5] and are heterogeneous in biogenesis, phospholipid composition, dimension, and load. The big family of EVs may generally include apoptotic bodies (ApoBDs), microvesicles (MVs), and exosomes. Both ApoBDs and MVs originate through outward blebbing and fragmentation of the plasma cell membrane, and they have dimensions usually ranging between 500 nm and 2 µm and from 50 nm to 1 µm, respectively. Exosomes, ranging in diameter from 30  to 120 nm, originate from the endocytic pathway and are enriched in endosome-associated proteins, such as annexins, Rab GTPases, and flotillin, and from plasma membrane proteins such as tetraspanins (CD9, CD63, and CD81) [6,7]. EVs are nowadays considered as effective mediators in intercellular communication, since during their biogenesis, the membrane and cargo composition can comprise specific nucleic acids, proteins, lipids, growth and angiogenic factors, transmembrane receptors, and extracellular matrix molecules that are able to modulate many physiological and pathological cellular processes [8]. Cell-derived vesicles can move a variety of cargo out of originating cells and deliver them to close or to remote cells and tissues. Extracellular vesicle transportation and networking can modulate immune reaction, tissue regeneration, tumor niche establishment, and tumor metastatization, effectively triggering phenotypic changes in acceptor cells, also without the delivery of their content [9,10]. This key role of EVs highlights their potentialities as vehicles for the intercellular delivery of therapeutic cargo molecules or as hybrid nanosized tools engineered ad hoc to regulate a physio-pathological condition or a disease progression. Of note is the innate capability of EVs to reach specific tissues. For example, tumor-derived exosomes have been proven to have a key role in tumor initiation, angiogenesis, and metastatization [11,12], a characteristic that is linked to the specific EVs’ composition and cell origin. In view of this homing attitude of EVs, many authors have reported on the capability of different cancer-cell-derived EVs (ccEVs) to be uptaken by parental cells. Albero at al. [13] showed that exosomes isolated from lung cancer A549 cells and then loaded with palladium exhibited tropism for their progenitor cells, discriminating over other cell types such as gliomas. Dumontel et al. reported that ZnO nanocrystals shuttled by extracellular vesicles, isolated from KB cells, were internalized by their parental cancer cells [14]. In a recent paper, Qiao et al. reported that HT-1080 fibrosarcoma-cell-derived exosomes can effectively home to the parent cell line that produced them [8]. Li et al. compared the autologous and heterologous cellular uptake of exosomes isolated from pancreatic and lung cancer cell lines, and the efficiency of autologous uptake was significantly higher than the heterologous one [15]. In vitro and in vivo studies on exosomes derived from murine colorectal and melanoma cancer cells, C26-Exos and B16BL6-Exos, respectively, showed that autologous C26-Exos were more effectively uptaken by C26 cells and were preferentially accumulated within C26 tumor tissue compared to the allogeneic counterpart [16]. Kim et al. compared the tropism of epithelial-cell-derived exosomes with cancer-derived exosomes, demonstrating an in vivo selective accumulation in the tumoral tissue of xenografted mice [17].

In contrast to the first type of tropism, recently reported in the contest of EVs’ “homing capability”, many in vitro and in vivo experiments showed a significant trafficking of EVs not limited to parental cells. The literature refers to heterologous tropism of tumor-derived EVs [18], supporting the theory that cancer cells can interact each other using exosomes and other types of EVs to promote metastatization [19,20]. Ji et al. reported that colorectal cancer cells (CRCs) release integrin beta-like 1-rich EVs in the bloodstream to activate fibroblasts of remote organs. These activated fibroblasts induce the formation of a pre-metastatic niche promoting metastasis, secreting pro-inflammatory cytokine such as IL-6 and IL-8 [21]. In addition to this mechanism, Shao et al. demonstrated that CRC-derived EVs are enriched with microRNA-21-5p, which is essential for the creation of a liver pro-inflammatory phenotype and metastasis [22]. Zheng et al. investigated the role of breast-cancer-derived EVs in metastatization. In detail, they observed the role of mitochondrial calcium uniporter in enhancing the angiogenesis of a metastatic niche due to the negative correlation with miR-4488 in breast-cancer-derived EVs [23].

Many authors reported that ccEVs also have targeting capabilities towards healthy cells. Some examples include the gastric-cancer-cell-derived exosomes in HUVEC cells that can induce angiogenesis enhancing tumor growth [24] or the release of tumor-derived EVs and their subsequent uptake by immune system, T, and NK cells. Such EVs can then inhibit and suppress the immune cell action, allowing the spreading of the tumor. This action has been recognized in EVs from melanoma cancer cells towards T cells [25] or from hypoxic tumors that can impair the antitumor immune response mediated by NK cells [26].

Last but not least, it should also be considered that healthy cell-derived EVs (hcEVs) can successfully be internalized by cancer cells, and this feature can be exploited for different therapeutic, even theranostic or clinical approaches [27,28,29,30].

Just trying to understand the role of EVs in in vitro and in vivo cell-to-cell communication, we could attempt to use these biological carriers, with or without engineering them, to develop new strategies applicable in the biomedical field. We evaluated which one of the two mechanisms of intercellular trafficking, homing, and targeting is the main phenomenon for our in vitro model. In particular, the targeting mechanism towards different cell lines was studied by post-engineering the lymphocyte-derived EVs with anti-CD20 monoclonal antibodies. In vitro tests were carried out on lymphocytes and on neoplastic human cell lines of myeloid (HL60) and lymphoid (Daudi) origin by using both native EVs (nEVs) and anti-CD20 (EVs^CD20^) engineered ones. Starting from the phenotypic characterization of both the cellular and EV membranes, the cytotoxic effect on cell viability was tested for 24 and 48 h of treatment with nEVs and EVs^CD20^. Suggestive images of fluorescence microscopy and further flow cytometry quantifications showed the high affinity between native lymphocyte EVs and the three cell lines, underlining how this type of hcEV is significantly internalized more by Daudi than by HL60. We also demonstrated how, by engineering the same type of EVs with a particular type of antibody (i.e., anti-CD20), it was possible to tune EV tropism towards the target cells. These studies, although preliminary, will soon allow the implementation of biological, synthetic, or hybrid vesicular systems for a wide range of nanotechnological applications in clinic theranostics.

## 2. Materials and Methods

### 2.1. Cell Cultures

All cell lines were cultured according to standard mammalian cell culture protocols and a sterile technique at 37 °C under a 5% CO_2_ atmosphere.

Daudi cells (ATCC^®^ CCL-213™), derived from a patient affected by Burkitt’s lymphoma, were obtained from American Type Culture Collection (ATCC). Cells were cultured in RPMI 1640 culture medium (ATCC) supplemented with 10% of heat-inactivated fetal bovine serum (FBS, ATCC), 1% penicillin/streptomycin (P/S, Sigma) in 75 cm^2^ non-treated cell culture flasks (Corning). The cell density was maintained of 3 × 10^5–6^ cells/mL.

The lymphocyte cell line (IST-EBV-TW6B) was purchased from the cell bank IRCCS AOU San Martino IST (Italy). Cells were grown in advanced RPMI 1640 culture medium (Gibco) with 20% heat-inactivated FBS (Gibco), 1% L-glutamine 200 mM (Lonza), and 1% P/S (Sigma) in 75 cm^2^ non-treated cell culture flasks (Corning) with a cell density of 9 × 10^4–5^ cells/mL.

HL60 cells (ATCC^®^ CCL-240™), derived from an acute myeloid leukemia patient, were obtained from ATCC. They were maintained in Iscove’s Modified Dulbecco’s Medium (Sigma) with 20% heat-inactivated FBS (Sigma), 1% glutamine (Sigma), 1% P/S (Sigma) in 75 cm^2^ non-treated cell culture flasks (Corning), adjusting the cell density to 1 × 10^5−6^ cells/mL.

### 2.2. Extracellular Vesicles Isolation

EVs were isolated from the 72 h conditioned media of the lymphocyte cell line maintained in advanced RPMI supplemented with 20% EV-depleted FBS, 1% glutamine, and 1% P/S. The depleted FBS was obtained by overnight centrifugation at 100,000× *g* (Type MLA-50 Rotor, Beckman Coulter) at 4 °C, and the supernatant was then used to complement the cell culture medium.

For EV production, 1.5 × 10^5^ lymphocytes/mL were maintained in a total volume of 200 mL of medium with depleted FBS in 75 cm^2^ non-treated flasks (Corning) for three days in vitro.

For each extraction, a cell viability >90% was requested to standardize the procedure and reduce the possibility of apoptotic bodies recovery. The EV extraction protocol was based on a sterile differential ultracentrifugation protocol, optimized by modifying the one described by Thery et al. [31]. In brief, the cell culture medium was collected and centrifuged at 150× *g* for 10 min at 4 °C to remove cells. The supernatant was then centrifuged at 2000× *g* for 20 min at 4 °C to remove the cell debris. The supernatant was again collected and centrifuged at 10,000× *g* at 4 °C for 30 min. Then, the supernatant was recollected, aliquoted into ultracentrifuge tubes (32 mL; OptiSeal Tubes, Beckman Coulter) and ultracentrifuged at 100,000× *g* for 70 min at 4 °C using an MLA-50 Rotor (OptiMax, Beckman Coulter). The resulting pellet was recovered, resuspended in cold 0.1 μm filtered phosphate buffered saline (PBS) solution, and centrifuged at 100,000× *g* for 60 min at 4 °C. The final pellet was recovered and resuspended in 600 µL of 0.1 μm filtered of physiological saline solution (0.9% NaCl; NovaSelect). Aliquots were stored at −80 °C.

### 2.3. Extracellular Vesicles Characterization

As recommended by the MISEV2018 guidelines, EVs were characterized following the different characterization steps [7]. The first one, the quantitative step, was accomplished by the total particle number and the total protein amount analysis, and since one of these components are exclusively associated with EVs, the ratio of particles:proteins was added to estimate the purity and, thus, the reliability of the quantity measures as reported in Webber at al. [32]. The second step, regarding the protein markers, was fulfilled by the immunophenotypical analysis of CD63 and CD81 markers and the third step regarding the single vesicle characterization by electron microscopy analysis.

The EVs’ concentration and size distribution were measured by Nanoparticle Tracking Analysis (NTA) with a NanoSight NS300 (Malvern Panalytical) equipped with a λ = 505 nm laser beam and a NanoSight syringe pump. All samples were resuspended in a final volume of 500 μL of physiologic solution to reach the correct particles/frame working value (from 20 to 100). All samples were measured by capturing three 60 s videos, with an infusion rate of 50 A.U. and the camera level ranging from 15 to 16. Collected videos were analyzed by the NTA 3.4 software with a detection threshold of 5.

EV membrane proteins were quantified using the colorimetric Bradford assay. To establish the calibration curve, a set of bovine serum albumin (BSA, Sigma) standards were prepared using PBS (0, 5, 10, 15, 20, 25, 40, 80, 100, and 160 μg/mL). The absorbance at 590 nm was read on a plate reader (Multiskan Go microplate spectrophotometer, Thermo Fisher Scientific, Waltham, MA, USA). The standards’ curve was plotted using a linear fitting, and the protein concentration of the samples was determined through the equation of the curve.

The purity of the EVs’ preparation was determined by calculating the ratio between particle number and protein concentration.

The EVs’ morphology was analyzed through transmission electron microscopy (TEM), with a Jeol JEM 1011 electron microscope using an acceleration voltage of 100 kV and equipped with a 2 Mp charge-coupled device (CCD) camera (Gatan Orius SC100). The EVs’ samples were diluted in physiological solution and vortexed for three minutes; then, a drop was deposited on a copper grid of 150 mesh, previously coated with an amorphous carbon film and plasma treated to remove hydrocarbon residues. The EVs’ sample was then stained, treating the sample grid with a 1% uranyl acetate solution in water for 30 s before the specimen dried.

For the analysis of the EVs’ markers, they were immobilized on aldehyde/sulfate latex beads, 4% *w*/*v*, 3 µm (Thermo Fisher), and analyzed by flow cytometry using the Guava easyCyte 6-2L flow cytometer (Merck Millipore). In brief, 5 μg of EVs measured by a Bradford assay was incubated with 10 μL of latex beads for 15 min at room temperature (RT). Then PBS was added to a final volume of 1 mL, and the solution was incubated for 2 h at RT. To saturate any free binding sites of the beads, 110 μL of PBS/1 M glycine were added and incubated for 30 min at RT. The samples were then centrifuged for 3 min at 4000 rpm, the supernatant was discarded, and the bead pellet was resuspended in 1 mL PBS/0.5% BSA. Beads were washed three times before incubation with CD63-PE (BioLegend), CD81-APC (BioLegend), CD20-PE (Miltenyi Biotec), and the respective isotype control. Unstained beads were employed to adjust instrument voltages and gate bead population to exclude debris and impurities derived from buffer solution. The 5 × 10^3^ gated events were acquired in a very low modality (0.12 μL/s flow rate). The PE signal was excited with a blue laser (488 nm) while the APC with a red laser (642 nm). The results were analyzed with Incyte Software in terms of the median fluorescence intensity (MFI) of the antigen minus the MFI of the isotype control [33,34], and histograms were plotted using the FCS Express 6 software. Each experiment was repeated five times (*n* = 5).

### 2.4. Cytofluorimetric Cell Membrane Markers Analysis

CD20, CD63, and CD81 surface expression was evaluated in lymphocytes, Daudi, and HL60 cells by flow cytometry using the Guava easyCyte 6-2L flow cytometer (Merck Millipore). For CD20 expression, 1.5 × 10^6^ cells were centrifuged at 300× *g* for 10 min and resuspended in 98 μL of PBS/0.5% BSA. Two microliters of the CD20 antibody solution (Miltenyi Biotec) or the respective isotype control (Miltenyi Biotec) were added to the suspension on ice and incubated for 10 min in the dark at 4 °C. Cells were then washed with 1 mL PBS/0.5% BSA and centrifuged again at 300× *g* for 10 min. For CD63 and CD81 surface expression, 1 × 10^6^ cells were centrifuged at 350× *g* for 5 min and resuspended in 95 μL of PBS/0.5% BSA with 5 μL of the CD63 or CD81 antibody solution (BioLegend) or the respective isotype controls (BioLegend), incubated for 15 min in the dark on ice. Cells were then washed twice with 1 mL PBS/0.5% BSA by centrifugation at 350× *g* for 5 min. All pellets were then resuspended in 1 mL of PBS/0.5% BSA and analyzed by flow cytometry, excluding debris signal. The antibody signal was read in the Yellow-B and Red-R channels with a flow rate of 0.59 μL/s. Experiments were repeated at least three times. The results are expressed in terms of MFI.

### 2.5. Extracellular Vesicle Labeling

Lymphocyte-derived EVs were labeled with Wheat Germ Agglutinin, Alexa Fluor™ 647 Conjugate (WGA647, Thermo Fisher, λex = 650 nm) for cytofluorimetric analysis or with Wheat Germ Agglutinin, Alexa Fluor™ 488 Conjugate (WGA488, Thermo Fisher, λex = 495 nm) for fluorescence microscopy analysis by adding 1 μL of WGA647 or WGA488 (concentration of the stock solutions: 0.1 mg/mL in PBS) of 100 μL of EVs in physiologic solution. After 30 min of incubation at 37 °C and 160 rpm, the labeled EV solution was filtered with 50 kDa Amicon Ultra 0.5 mL centrifugal filters (Merck Millipore, Burlington, MA, USA) to remove the unbound dye.

### 2.6. EV^CD20^ Nanoconstruct Preparation

**Figure 1:**
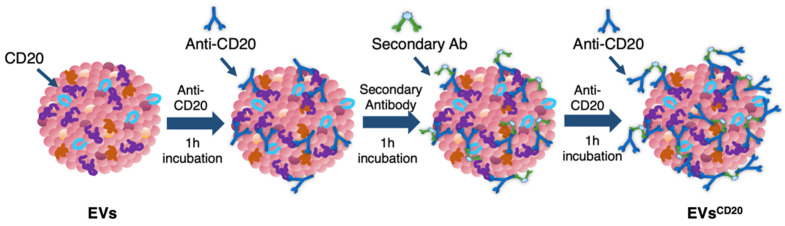
Schematic representation of the three steps for EV surface functionalization with the targeting antibody anti-CD20.

Since the lymphocyte-derived EV membrane expresses the CD20 antigen as well as the membrane of the considered cell lines, a sandwich multistep functionalization was provided in order to label the lymphocyte-derived EV membrane with anti-CD20 monoclonal antibodies oriented for the targeting (Figure 1). The EV^CD20^ was prepared starting from with an EV aliquot with 5 μg/mL of protein content in physiologic solution. For the microscopy and cytofluorimetric analysis, EV membrane was previously labeled as described above. Labeled EVs and non-labeled aliquots (for cytotoxicity experiments) were filtered with 50 kDa Amicon filters to remove the unbounded dye and/or concentrate the solution; then, the eluate was resuspended in 0.1 μm filtered PBS. To promote coupling with the CD20 antigen, in the first step of the functionalization process, an excess amount of anti-CD20 monoclonal antibody (Rituximab, Anti-Human CD20 Therapeutic Antibody, 1 mg/mL in PBS, Creative Biolabs) was used, as well as considering CD20 antigen at least half of the total protein amount. Thus, anti-CD20 in a molar ratio of 4:1 (anti-CD20:CD20) was added to the EVs’ solution and incubated for 1 h at RT on a tube-rotator with a fixed speed of min^−1^. Then, a molar ratio of 1:1 (secondary antibody:anti-CD20) of anti-human secondary antibody (AffiniPure F(ab’)₂ Fragment Goat Anti-Human IgG, Fcγ fragment specific, 1.3 mg/mL in water, Jackson Immunoresearch) was added and incubated on the tube rotator for 1 h at RT. The last step was carried out by adding the same amount of anti-CD20 in the first step and incubating for another hour. After the three conjugation steps, the sample was purified by ultrafiltration with 50 kDa Amicon filters, and the eluate was resuspended in cell medium for the cells’ treatments. For the preparation of the nEV control sample, the anti-CD20 antibody was replaced with PBS buffer and the secondary antibody with bidistilled water.

### 2.7. Cytotoxicity Assay of nEVs and EVs^CD20^

To test the cytotoxicity of nEVs and EVs^CD20^ in lymphocytes, Daudi, and HL60 cell lines, EVs were concentrated by ultrafiltration using 50 kDa Amicon filters. For nEVs, the eluted solution was resuspended in cell culture medium to reach the defined volume of EV solution.

To evaluate the viability of nEVs and EVs^CD20^ in lymphocytes, Daudi, and HL60 cell lines, 2 × 10^5^ cells for each mL of treatment were centrifuged, and the supernatants replaced with the treatment solutions with 0, 5, 10, and 20 μg/mL of nEVs or 5 μg/mL of EVs^CD20^. A total volume of 100 μL was plated for each well in a 96-well flat-bottom plastic culture plate (Greiner Bio-One, 96 Well for suspension culture). After 20 and 44 h of incubation, 10 μL of WST-1 reagent (CELLPRO-RO Roche) was added to each well, and after a further 4 h of incubation, the formazan absorbance was detected at 450 nm by the Multiskan Go microplate spectrophotometer (Thermo Fisher Scientific Waltham, MA, USA) using a 620 nm reference. All experiments were carried out at least in triplicate for each cell line, and the results were normalized to the control.

### 2.8. Cytofluorimetric Analysis of nEV and Es^CD20^ Internalization

For the uptake evaluation of nEVs, the vesicles were labeled with WGA647 and resuspended in cell medium to return to the final concentration of 5, 10, and 20 μg/mL. The uptake of EVs^CD20^ was evaluated at the concentration of 5 μg/mL of EV protein content.

To evaluate the internalization of nEVs and EVs^CD20^ in lymphocytes, Daudi, and HL60 cell lines, 2 × 10^5^ cells for each mL of treatment were centrifuged, and the pellets were resuspended in the treatment solutions. The experiment was carried out twice for nEVs and three times for EVs^CD20^ for each cell line. Data from untreated cells were used as reference. Cells of both native and targeted EV experiments were cultured in a non-treated 24-well plate (Thermo Scientific, Waltham, MA, USA), 500 μL for each well. After 24 and 48 h of incubation, the contents of the different wells were collected and washed twice in PBS at 130 g for Daudi and HL60 and 150 g for lymphocytes and resuspended in 350 μL of PBS for the 24 h and 500 μL for the 48 h cytofluorimetric analysis. The 1 × 10^4^ events were counted with the flow cytometer with a 0.59 μL/s flow rate, excluding cell debris. The analyses were performed using a red laser (λ_ex_ = 642 nm). The positive events were characterized by a shift in Red-R fluorescence intensity (emission filter 661/15) and the percentages of positive events were compared to untreated cells and evaluated with Guava InCyte Software (Merck Millipore).

### 2.9. Fluorescence Microscopy Imaging of nEV and EV^CD20^ Internalization

For fluorescence microscopy analysis, nEVs were labeled with WGA488 and the samples were treated with the same protocol used for the cytofluorimetric analysis. After 24 and 48 h of culturing at 37 °C and 5% CO_2_ in 24-well plates, the content of each well was collected, centrifuged at 130× *g* for Daudi and HL60 and 150 g for lymphocytes, and resuspended in 50 μL of the correspondent medium. The 50 μL cell solution was spotted in a single drop in the center of a well of a 4-well chamber slide (Thermo Scientific™ Nunc™ Lab-Tek™ II CC2™ Chamber Slide System, Waltham, MA, USA) and placed at 37 °C and 5% CO_2_ for 30 min to allow the seeding of the cells. Next, each well was brought up to a final volume of 500 μL with cell medium and 2.5 μL of WGA647 (concentration of the stock solution: 1 mg/mL in PBS) were added to label cell membranes. After 5 min of incubation at 37 °C, 0.25 μL of Hoechst (Thermo Fisher, λ_ex_ = 361 nm) was added to each well to stain DNA and nuclei, and after further 5 min of incubation, two washing steps were performed using 500 µL of Live Cell Imaging solution (LCI, 1X, Molecular Probes) at 37 °C.

For the fluorescence microscopy analysis of EVs^CD20^, EVs were initially labeled with WGA488 and then the EVs^CD20^ were prepared as described in the dedicated section using a fluorescent anti-human secondary antibody (AMCA-AffiniPure F(ab’)₂ Fragment Goat Anti-Human IgG, Fcγ fragment specific, 1.5 mg/mL in water, Jackson Immunoresearch, λ_ex_ = 350 nm). Samples were treated with the same protocol used for the cytofluorimetric analysis in a 96-well plate (Greiner Bio-One, 96 Well for suspension culture), 250 μL for each well. After 24 and 48 h of culturing, the content of each well was collected and seeded as described above for nEVs in an 8-well chamber slide (Thermo Scientific™ Nunc™ Lab-Tek™ II CC2™ Chamber Slide System, Waltham, MA, USA) to a final volume of 250 μL. Only cell membranes were labeled using WGA647, and the microscopy imaging was performed.

Live cell imaging analyses were carried out using an incubator gas chamber (Okolab) equipped with CO_2_ sensors, a temperature unit, and an active humidity controller to maintain cells in their physiological conditions, and images were acquired using a wide-field fluorescence-inverted microscope (Eclipse Ti-E, Nikon, Tokyo, Japan) equipped with a super bright wide-spectrum source (Shutter Lambda XL), a high-resolution camera (Zyla 4.2 Plus, 4098 × 3264 pixels, Andor Technology, Belfast, UK) using an immersion oil 100× objective (1.3 Apo, Nikon). The orthogonal views were obtained on Z-stack images using the specific tool in the NIS-Element software (NIS-Elements AR 4.5, Nikon).

### 2.10. Statistical Analysis

Plotted data are the mean ± standard error or the mean ± standard deviation. The statistical analysis between the treatment groups was performed using two- or three-way analysis of variance (ANOVA) tools in the SIGMA Plot software’s data analysis package. ** *p* < 0.001 and * *p* < 0.05 were considered significant. Independent experiments were performed at least two times.

## 3. Results

### 3.1. Extracellular Vesicle Characterization

**Figure 2:**
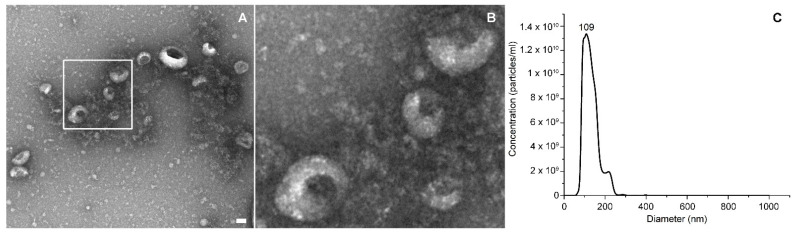
Panel (**A**) represents a TEM image of EVs and (**B**) a higher magnification of the vesicles in the white box. Scale bar: 50 nm. In panel (**C**), there is an NTA representative size distribution graph.

TEM images in Figure 2A,B showed a heterogeneous population of extracellular vesicles, among which exosomes were recognizable from their typical cup shape.

Different EVs’ isolations were characterized with NTA and Bradford assays. The results derived from 15 different extraction sessions were considered. The average concentrations reported by the NTA measurements was 1 × 10^11^ ± 6 × 10^10^ part/mL, while an example of the size distribution of lymphocyte-derived EVs is provided in Figure 2C; the protein content, measured by Bradford assay on the same isolations, was 140 ± 36 μg/mL, and the EVs’ purity was 8 × 10^8^ ± 3 × 10^8^ part/μg, all expressed as the mean ± SD. The EVs’ purity calculation only provided an indication regarding the realistic purity estimation of the sample, since it could be affected by the presence of soluble proteins or protein aggregates. An image of WGA488-labeled EVs are reported in Figure S1 in the Supplementary Materials.

### 3.2. Extracellular Vesicles and Cell Surface Marker Analysis

**Figure 3:**
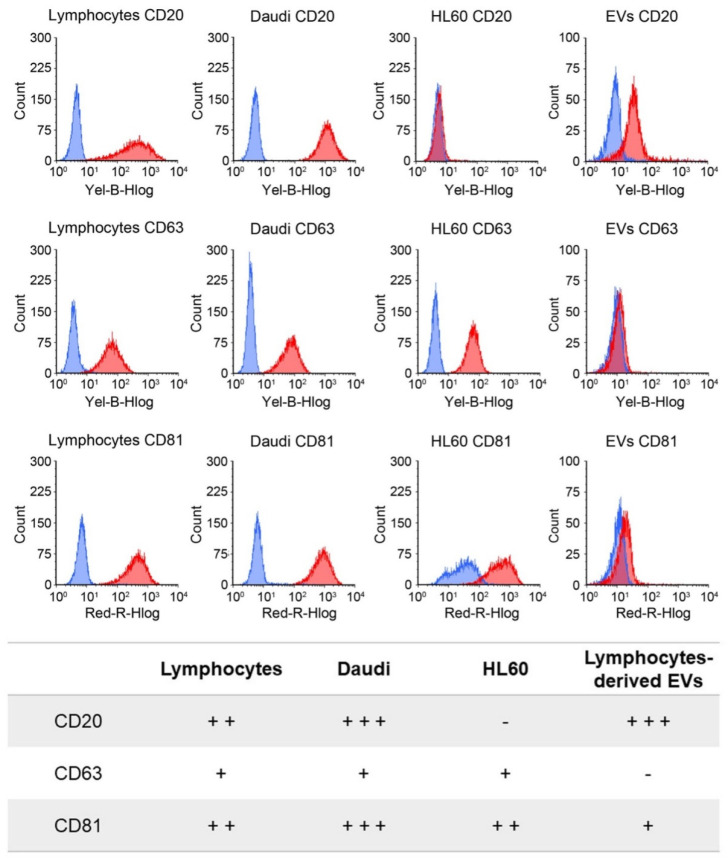
Cytofluorimetric evaluation of CD20, CD63, and CD81 marker expression levels on the plasma membrane of the three different cell lines and on the lymphocyte-derived EV membranes. In the upper panel, representative histograms of the expression of the different markers are reported for lymphocytes, Daudi, HL60, and EVs (blue represents the isotype controls, and red represents the related tested antigen). In the lower panel, the expression levels are grouped into negative (–), low (+), intermediate (++), and high (+++) categories, corresponding to the following signal intensities: <10, 10–100, 100–500, and 500–1,000, respectively, for cells and <1.5, 1.5–5, 5–10, and 10–20 for EVs.

In order to appreciate the tropism and related targeting capability of native and engineered B-lymphocyte EVs, we verified the CD20, CD63. and CD81 antigens’ expression both on the cells’ and EVs’ membranes (Figure 3). Considering the cellular plasma membrane, the CD20 antigen was strongly expressed in Daudi, moderately in lymphocytes, and was almost absent in HL60 cells (*p* ≤ 0.001 for Daudi vs. HL60 and lymphocytes, *p* = 0.019 for lymphocytes vs. HL60). CD63 was expressed slightly similarly by the three cell lines, while the CD81 level was high, in particular on Daudi cell membrane (*p* ≤ 0.001 for Daudi vs. lymphocytes and *p* = 0.003 for Daudi vs. HL60).

Concerning the EVs, the CD20 marker expression level was significantly higher than for CD63 and CD81 (*p* ≤ 0.001), since they were lowly expressed or almost absent on lymphocyte-derived EV membranes.

In this work, only transmembrane proteins associated to plasma membrane were investigated, while cytosolic proteins and purity markers, such as components of non-EV co-isolated structures, were not evaluated.

### 3.3. Cytotoxicity Assay of Treatment with Native Lymphocyte-Derived EVs

**Figure 4:**
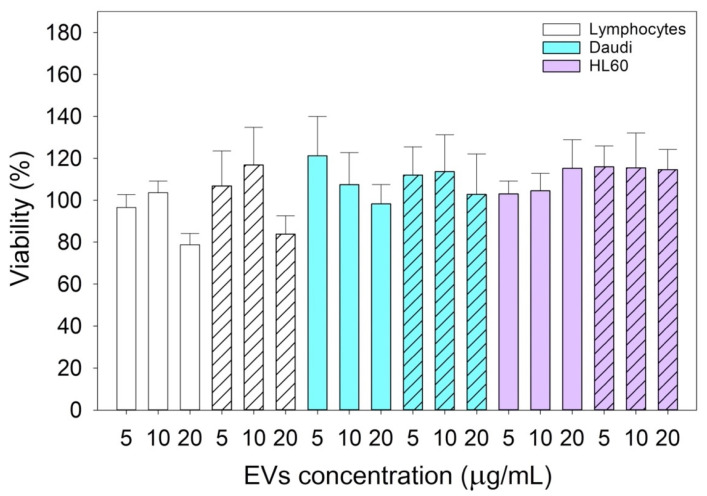
Cytotoxicity assay results of lymphocytes, Daudi, and HL60 cell lines treated for 24 (solid color bars) and 48 (dashed bars) hours with 5, 10, and 20 μg/mL of concentrated nEV suspensions. Plotted data are the mean ± SE. The comparisons between cell lines, times, and types of treatments were performed using the three-way ANOVA test, and no statistically significant differences resulted. Independent experiments were carried out three times.

In Figure 4, the cytotoxicity assay results showed that the treatments had no effect on cell viability since they were like the controls. There were no statistically significant differences considering both 24 and 48 h treatments on the three different cell lines with 5, 10, and 20 μg/mL of native lymphocyte-derived EV suspensions.

### 3.4. Cytofluorimetric Analysis of nEVs’ Uptake

**Figure 5:**
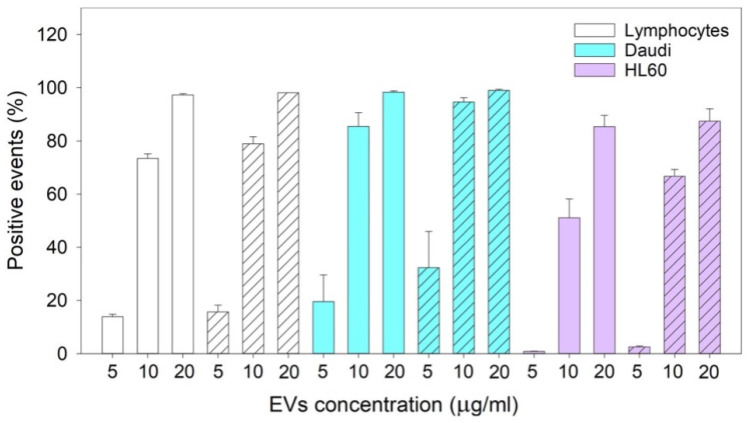
The graph bar represents the mean and the SE of the results of nEVs’ uptake in lymphocytes, Daudi, and HL60 cell lines at different concentrations (5, 10, and 20 μg/mL) at 24 (solid color bars) and 48 (dashed bars) hours. Plotted data are the mean ± SE. The comparisons between cell lines, times, and types of treatments were performed using the three-way ANOVA test, and the statistical significance is reported in the main text. Independent experiments were carried out two times.

Three-way ANOVA and multiple comparison procedure on data collected for two different experiments (Figure 5) showed that the uptake of nEVs, extracted from lymphocyte cell cultures, did not vary among the three cell lines depending on the administration time (24 and 48 h). On the contrary, nEV uptake was significantly different considering the concentrations at which the treatments were carried out, both within the same cell line and between the different cell types.

When considering the same cell line and concentration, there was an increasing nEV uptake that depended on the administration time; there was a significant difference between 24 and 48 h (*p* = 0.003). Meanwhile, by increasing the concentration of nEVs, the nEVs’ internalization also increased; there was a noticeable boost in the uptake between 5 and 20 μg/mL (*p* ≤ 0.001 for all the cell lines), 5 and 10 μg/mL (*p* ≤ 0.001 for all the cell lines), 10 and 20 μg/mL (*p* ≤ 0.001 for lymphocytes and HL60 and *p* = 0.02 for Daudi). Comparing the different cell lines at the same concentration resulted in the uptake of nEVs being considerably different among all the cell lines at 5 μg/mL (*p* ≤ 0.001 Daudi vs. HL60, *p* = 0.005 Daudi vs. lymphocytes, and *p* = 0.003 lymphocytes vs. HL60) and 10 μg/mL (*p* ≤ 0.001 for all the comparisons), while at 20 μg/mL, the nEVs’ uptake from HL60 was significantly lower than the one from lymphocytes and Daudi (*p* = 0.01 for both HL60 vs. lymphocytes and Daudi). On the contrary, the nEVs’ uptake was similar when comparing lymphocytes with Daudi (*p* = 0.8).

### 3.5. Fluorescence Microscopy Assay of nEVs’ Uptake

**Figure 6:**
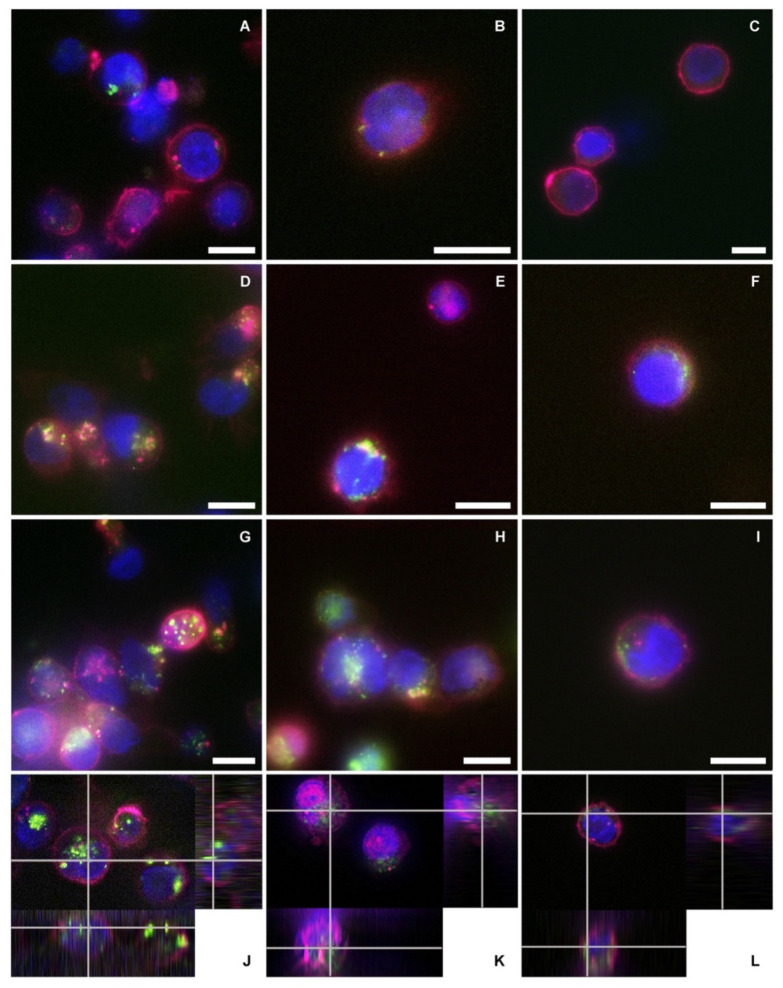
Schemes followed the same formatting. Fluorescence microscopy images of the three cell lines treated with different concentrations of lymphocyte-derived EVs after 48 h of incubation. The blue color represents the cells’ nuclei labeled with Hoechst, purple depicts the cells’ membranes labeled in WGA647, and green shows the nEVs labeled in WGA488. Images of (**A**,**D**,**G**) lymphocytes, (**B**,**E**,**H**) Daudi, and (**C**,**F**,**I**) of HL60 cell lines incubated with a concentration of 5, 10, and 20 µg/mL of nEVs, respectively. Orthogonal views of the internalization of 20 µg/mL nEVs in lymphocytes (**J**), Daudi (**K**), and HL60 (**L**) treated for 48 h. Scale bars: 10 µm.

Fluorescence microscopy images qualitatively confirmed the cytofluorimetric assay results (Figure 6). There was an evident increase in the nEVs’ internalization related to the concentration used to treat the three different cell lines. Images of treatments after 48 h of incubation, performed using 5 μg/mL (A, B, and C for lymphocytes, Daudi, and HL60, respectively) showed a reduced nEV internalization compared to images of treatments conducted with 10 μg/mL (D, E, and F) and 20 μg/mL (G, H, and I). In panels J, K, and L, the representative orthogonal views of nEVs at 20 μg/mL highlighted the presence of the vesicles inside the three tested cell lines, not only adherent to the plasma membranes.

### 3.6. Cytotoxicity Assay of EVs^CD20^

**Figure 7:**
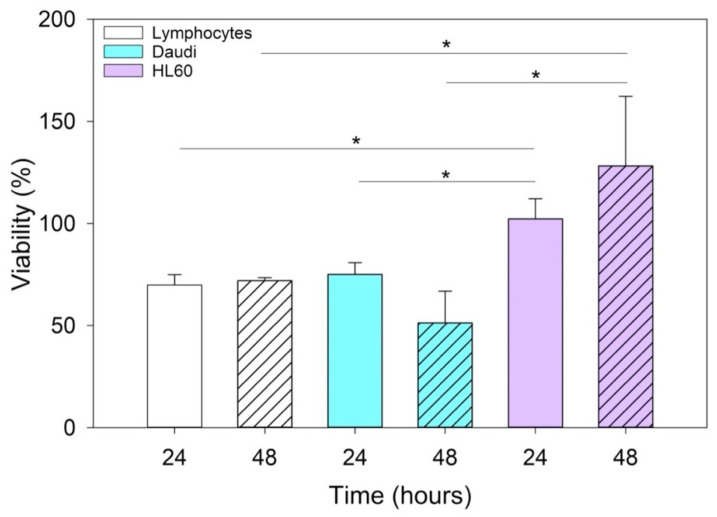
Histograms represent the mean and the SE of the EV^CD20^ viability data in lymphocytes, Daudi, and HL60 cell lines treated with 5 μg/mL of EVs^CD20^ solution for 24 and 48 h. The comparisons between cell lines and times of incubation were performed using two-way ANOVA; * *p* < 0.05. Independent experiments were performed three times.

After confirming by fluorescence microscope that the EVs^CD20^ nanoconstruct was successfully prepared (Supplementary Materials Figure S2), cellular tests were carried out. The results of the cytotoxicity assay of cells treated for 24 and 48 h with 5 μg/mL of EVs^CD20^ are shown in Figure 7. The viability values of the lymphocytes and Daudi cell lines were significantly reduced by the treatment in contrast to the HL60 cells.

Since anti-CD20 monoclonal antibodies actually represent the gold standard treatment of B-cell hematological malignancies [35,36,37], we expected an increase in cytotoxicity with the use of vesicles engineered with anti-CD20. In particular, this effect was more marked for lymphocytes and Daudi than for HL60, because they expressed the CD20 antigen on their surfaces. Furthermore, many in vitro studies have demonstrated that the binding of the anti-CD20 monoclonal antibodies can trigger cell death also without immune system effector mechanisms [38,39,40].

### 3.7. Cytofluorimetric Assay of EV^CD20^ Uptake

**Figure 8:**
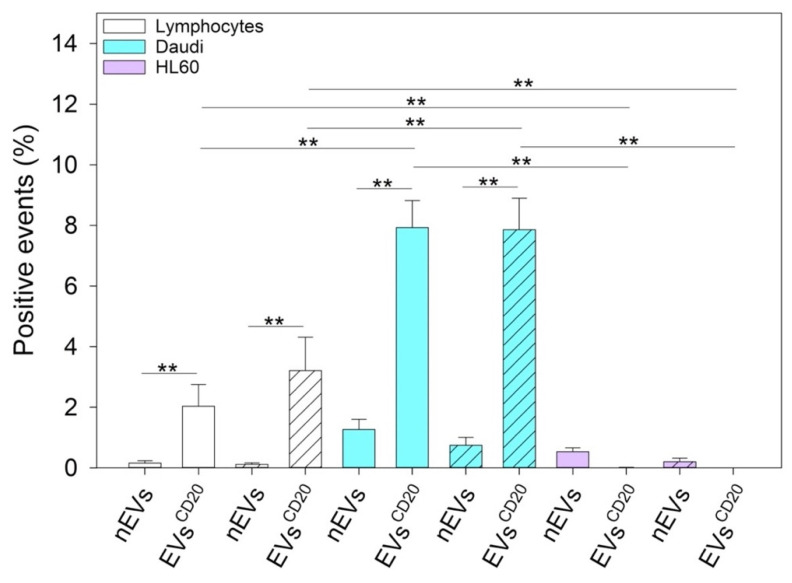
The graph represents the average and the SE of the results of targeted lymphocyte-derived EV uptake in lymphocytes, Daudi, and HL60 cell lines at a 5 μg/mL concentration at 24 (solid color bars) and 48 (dashed bars) hours. The table shows the resumed three-way ANOVA analysis of the results; ** *p* < 0.001. Independent experiments were performed three times.

Three-way ANOVA and multiple comparison procedures on data collected from three different independent experiments showed that lymphocytes and Daudi cellular uptake of EVs significantly increased by treating cells with anti-CD20 targeted EVs compared to using native EVs (Figure 8). Fully supported by the evidence that HL60 does not express CD20 on their plasma membranes, the uptakes of nEVs and EVs^CD20^ in HL60 were not statistically different.

### 3.8. Fluorescence Microscopy Assay of EV^CD20^ Uptake

**Figure 9:**
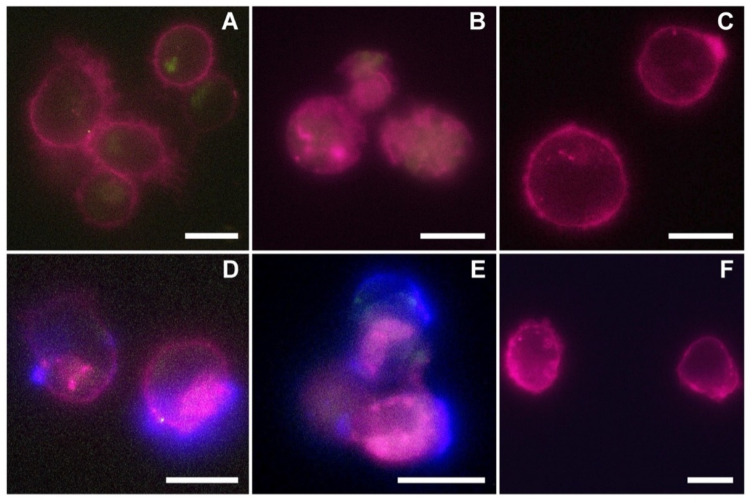
Representative fluorescence microscopy images of the three cell lines incubated for 48 h with 5 μg/mL of native (top panels) and anti-CD20 engineered lymphocyte-derived EVs (bottom panels). Panels A and D refer to lymphocytes, B and E to Daudi, and C and F to HL60 cell lines incubated with nEVs (**A**–**C**) and EVsCD20 (**D**–**F**). The purple color represents the cells’ membrane labeled with WGA647, green refers to the EVs labeled in WGA488, while the blue channel refers to the AMCA fluorescent signal of the secondary antibody linked to the nanoconstruct. Scale bars: 10 µm.

In Figure 9, the fluorescence microscopy images of native (A, B, and C) and targeted (D, E, and F) EVs at 5 μg/mL after 48 h of incubation were reported for the three cell lines (lymphocytes: A and D; Daudi: B and E; HL60: C and F).

Fluorescence images qualitatively confirmed cytofluorimetric analysis. Panels A, B show a low internalization of the nEVs in the cells’ cytosolic compartment in respect to the lymphocytes (D) and Daudi (E) treated with EVs^CD20^. In more detail, the evident blue fluorescence referring to the surface functionalization of EVs^CD20^ underlined the efficacy of the active tropism induced by the proposed bioengineering using the CD20 antibody. In respect to the lymphocytes and Daudi cell lines, the HL60 cells, not expressing CD20 markers on their plasma membrane, showed no internalization enhancement deriving from the use of EVs^CD20^, and the uptake of both native and targeted EVs was almost absent (panels C and F, respectively).

## 4. Discussion

EVs are currently considered as effective mediators in intercellular communication [8]. Cell-derived vesicles can indeed transport different cargo (i.e., mainly proteins and nucleic acids) out of originating cells and deliver them both to close and distant cells and tissues. EVs thus show a key role in the regulation of many physiological and pathological conditions related to cell growth, regeneration, and immune system response. This behavior highlights the powerful potential of EVs as vehicles for the intercellular delivery of both natural components or even artificially and ad hoc inserted cargo. This idea thus provides a solid groundwork for the design and engineering of new drug delivery solutions and hybrid nanotechnological devices based on EV modification in terms of both cargo and/or membrane composition, enhancing their transport and tropism towards recipient cells [4,14].

Many studies have actually showed that surface proteins at the EV membrane, such as tetraspanins, integrins, and immunoglobulins, participate in the uptake of EVs. The cell internalization of EVs can take place through the fusion of plasmatic or endosomal membrane, micropinocytosis, phagocytosis, and clathrin-mediated endocytosis. In this contest, the functions of precise protein–protein and lipid rafts interactions have been investigated to assess the role of specific antibodies or of chemical inhibitors able to interfere with specific uptake paths. Furthermore, it is relevant to consider when developing EVs as nanoengineered carriers that EVs can transfer information to target cells either without the release of their cargo (i.e., by approaching the cell’s surface as for immune reactions) or after they have been internalized [10].

Starting from the fact that EVs are biocompatible, low-immunogenic efficient loaders and carriers of a series of different molecules, they can be considered as excellent candidates for being engineered and used in a whole series of promising nanotechnological applications [4]. EVs can be internalized more than artificial liposomes because they usually show a higher specificity for tumoral cells, and their size allows them to reach tumoral tissues, taking advantage of the enhanced retention and permeability effects [41,42].

The trafficking ability of EVs can be influenced by different factors. Their protein content can drive them towards specific compartments, and the lipid profile can influence their uptake by different cell types. EVs rich in integrin α6 in complex with β1 and β4 subunits are conducted preferentially towards fibroblast and epithelial cells in lung, while, when in complex with β5 and β4 subunits, towards the Kupffer cells in the liver and toward CD31-positive endothelial cells in the brain [43]. EVs rich in integrin α4 and tetraspanin Tspan8 selectively target pancreatic cells [44], while CD63-positive EVs are directed to neuronal and glial cells and the CD63-negative EVs to neuronal dendrites [45]. Fibronectin drives microvascular endothelial-cells-derived EVs towards oligodendrocyte precursor cells [46].

The presence of phosphatidylserine and glycans in their membrane selectively drives their uptake by macrophages or mesenchymal stem cells [47,48,49].

More in general, considering the directionality of their intercellular trafficking, EVs are characterized by evident “homing” and “targeting” ability, displaying, in the first case, tropism for parental cells, while approaching and docking to different cells types or tissues in the second one.

In view of these two possible mechanisms, we wished to explore with in vitro experiments the significant trafficking of EVs isolated from B lymphocytes not limited to healthy parental cells. We then evaluated their innate tropism towards two different types of hematological cancer cells (Burkitt lymphoma, Daudi, and human myeloid leukemic cells, HL60). Most interestingly, here we also proposed antibody-engineered EVs and investigated the possibility of selectively targeting the Daudi cell line.

The reported cytotoxicity assay results, uptake, and fluorescent microscopy analyses (Figure 4, Figure 5 and Figure 6) clearly show the nEVs’ biocompatibility and innate tropism towards the tested cell lines, highlighting a significantly higher internalization of nEVs in both B lymphocytes and Daudi cell lines with respect to HL60 cells.

To distinguish between the innate capability of the passive targeting of the nEVs and active heterologous targeting, we then engineered the EVs with monoclonal antibodies, anti-CD20, producing EVs^CD20^. The rationale of this choice lies on the preliminary phenotypic characterization of the antigen expression at the cell membrane performed on B lymphocytes, Burkitt lymphoma, and human myeloid leukemic cells. The result of this analysis, shown in Figure 3, reports a relevant CD20 biomarker expression in Burkitt lymphoma cells and slightly lower in B lymphocytes, while CD20 expression was almost absent in human myeloid leukemic cells. We have therefore shown a significant ability to selectively drive such engineered EVs^CD20^ and enhance their internalization events into the Daudi cancer cell line as highlighted in Figure 8.

Since this class of vesicles is very heterogeneous and easily available, both from in vitro cell cultures and from the biological fluids of patients, they have high potential in the diagnosis and treatment of an increasingly broad category of diseases. Our in vitro experimental results showed that EVs isolated from healthy cells, such as B lymphocytes, were fully tolerated in terms of cytotoxicity in different in vitro systems, whether healthy or pathological (Figure 4). At the same time, according to the confirmation of their high biocompatibility, the verification of their exceptional tropism was carried out in terms of homing towards the parental cell line and towards other cell lines, taken here as model systems (Figure 5). Furthermore, the antibody post-extraction modifications here described have shown how the EVs allow a whole series of chemical–physical and biological engineering which, although slightly worse tolerated in terms of biocompatibility (Figure 7), have been shown to be effective in increasing EVs’ targeting active tropism towards specific cell lines, organs, or tissues (Figure 8).

Although this method used to functionalize the surface of lymphocyte-derived EVs presents some drawbacks that must be improved, for example, the low yield, the scalability, and the high cost, there are many advantages. The first one is the biological strategy used for the functionalization; in fact, avoiding physical approaches, such as sonication, extrusion, and freeze–thaw, or chemical methods, such as click-chemistry, amidation, or functional group insertions, have allowed the addition of a further functionality to the EVs, preserving their membrane integrity [50]. The proposed surface functionalization through targeting antibody followed a new engineering approach for EVs, which began to spread over the last years, for a new class of cell-free cancer therapy. Furthermore, in contrast to some applications, such as SMART-Exos, which target T-cell surface CD3 [51], and EXO-DEPTs, which are directed towards the HER2 receptor of breast cancer [52], this approach is simpler because it does not require the transfection of the parental cells, but it is a post-isolation method of functionalization such as the one described by Kooijmans et al. [53].

Last but not least, this proposal is a definitely winning approach in the field of personalized therapy. By engineering EVs isolated from patients’ biological fluids, with a very wide range of proteins or small peptides, its effectiveness and biocompatibility are certainly increased compared to the same solutions implemented with exogenous or synthetic material.

## 5. Conclusions

To conclude, the above reported in vitro studies provided a rationale for understanding and distinguishing between innate tropism of nEVs and the targeting capability of antibody-engineered EVs.

Through phenotypic characterization, cytotoxicity assays, uptake, and fluorescent microscopy analyses, we evaluated nEVs’ biocompatibility and their natural tropism capability, showing a significantly higher internalization of nEVs in both B lymphocytes and Daudi cells.

To distinguish between the innate capability of passive targeting of the nEVs and the active heterologous targeting, we engineered the EVs with an anti-CD20 monoclonal antibody producing EVs^CD20^. We successfully verified a significant targeting ability of engineered EVs^CD20^ capable of selectively driving them and enhancing their internalization events into Daudi cancer cells.

In conclusion, these studies will pave the way for the implementation of new vesicular systems that can be engineered with a wide range of monoclonal antibodies. In a full personalized medicinal context, direct isolation from the patients’ body fluids could promote the customization of biomimetic nanodevices characterized by an outstanding biocompatibility for further therapeutic, diagnostic, or even theranostic applications.

## Data Availability

All data are reported in the present manuscript and not elsewhere.

## Supplementary Materials

The following are available online at https://www.mdpi.com/article/10.3390/membranes11110886/s1, Figure S1: Evidence of nEVs labelling, Figure S2: EVsCD20 characterization.
