# Supplementary material for: Extracellular Vesicles Tropism: A Comparative Study between Passive Innate Tropism and the Active Engineered Targeting Capability of Lymphocyte-Derived EVs
Source: Membranes (Basel). 2021 Nov 18;11(11):886. doi: 10.3390/membranes11110886 (PMC8617986; doi:10.3390/membranes11110886)
Supplement: Supplementary file 1 [file membranes-11-00886-s001.zip › membranes-1464888-supplementary.pdf]

Supporting Information

Extracellular Vesicles Tropism: A Comparison study between Passive Innate tropism versus Active Engineered Targeting Capability of lymphocyte derived-EVs

Tania Limongi [†,] *, Francesca Susa [†], Bianca Dumontel, Luisa Racca, Michela Perrone Donnorso, Doriana Debellis, and Valentina Cauda*

Evidence of nEVs labelling

The fluorescence microscope images of WGA-labelled nEVs are reported in Figure S.1, proving the labelling efficiency. nEvs were labelled with Wheat Germ Agglutinin, Alexa Fluor™ 488 Conjugate (WGA488, Thermo Fisher, λex=495 nm) as described in the Materials and Method section of the main manuscript in Section 2.5.

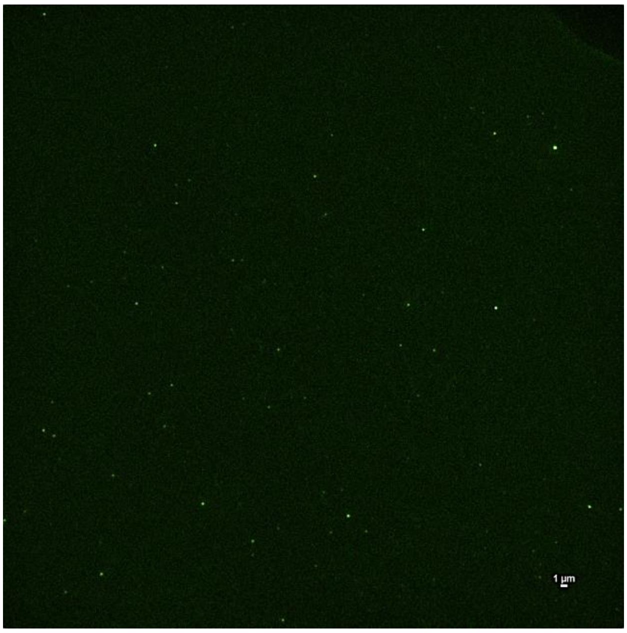

Figure S1. Fluorescence microscopy image on nEV labelled with WGA488, imaged in the green channel. Scale bar is 1 µm.

EVs^{CD20} characterization

The EVs^{CD20} characterization was performed also by fluorescence microscopy. Figure S2 reports an image of the EVs^{CD20} nanoconstruct where the EVs are initially labelled with WGA488 (λex=495 nm) and then the EVs^{CD20} labelled using a fluorescent anti-human secondary antibody (AMCA-AffiniPure F(ab')$_2$ Fragment Goat Anti-Human IgG, Fcγ fragment specific) conjugated to coumarin dye (λex=350 nm). The sample is then imaged after depositing it on a microscope glass slide covered by a cover glass slip. The preparation method is described in Section 2.9 of the main paper.

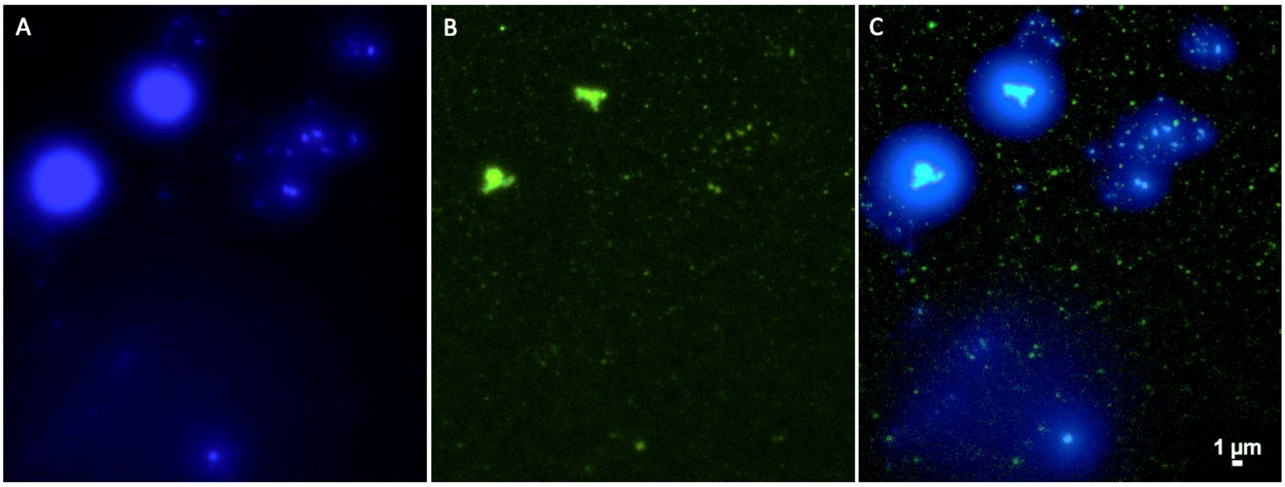

Figure S2. Fluorescence microscopy images of the **EVsCD20.** A is the blue channel of the secondary antibody of the antibody functionalization, B is the green channel of EVs' membranes and C is the merged image. Scale bar is 1 µm and the same for each channel picture.
